# [^18^F]Fluorothymidine Uptake in the Porcine Pancreatic Elastase-Induced Model of Abdominal Aortic Aneurysm

**DOI:** 10.3390/jimaging7080130

**Published:** 2021-08-04

**Authors:** Richa Gandhi, Joanna Koch-Paszkowski, Charalampos Tsoumpas, Marc A. Bailey

**Affiliations:** Leeds Institute of Cardiovascular and Metabolic Medicine, University of Leeds, Leeds LS2 9JT, UK; richa.gandhi@utoronto.ca (R.G.); j.koch-paszkowski@leeds.ac.uk (J.K.-P.); c.tsoumpas@leeds.ac.uk (C.T.)

**Keywords:** positron emission tomography, aneurysm, mice, radioactive tracer, abdominal aorta

## Abstract

The porcine pancreatic elastase (PPE) model is a common preclinical model of abdominal aortic aneurysms (AAA). Some notable characteristics of this model include the low aortic rupture rate, non-progressive disease course, and infra-renal AAA formation. Enhanced [^18^F]fluorothymidine ([^18^F]FLT) uptake on positron emission tomography/computed tomography (PET/CT) has previously been reported in the angiotensin II-induced murine model of AAA. Here, we report our preliminary findings of investigating [^18^F]FLT uptake in the PPE murine model of AAA. [^18^F]FLT uptake was found to be substantially increased in the abdominal areas recovering from the surgery, whilst it was not found to be significantly increased within the PPE-induced AAA, as confirmed using in vivo PET/CT and ex vivo whole-organ gamma counting (PPE, *n* = 7; controls, *n* = 3). This finding suggests that the [^18^F]FLT may not be an appropriate radiotracer for this specific AAA model, and further studies with larger sample sizes are warranted to elucidate the pathobiology contributing to the reduced uptake of [^18^F]FLT in this model.

## 1. Introduction

An abdominal aortic aneurysm (AAA) is an asymptomatic, localised dilatation of the abdominal aorta larger than 30 mm in maximal diameter that progresses to rupture, leading to profound internal bleeding and mortality. Patients with AAA are typically diagnosed incidentally or through ultrasound scanning (USS)-based screening programmes that are used to make surgical decisions and predict the risk of mortality. However, anatomical size alone may be insufficient as an indicator of prognosis. Positron emission tomography (PET)/computed tomography (CT) is posited to be useful for patient risk stratification and represents an active field of study in the context of AAA [1,2,3].

Much of our understanding of AAA pathophysiology is derived from effective preclinical models that aim to mimic key features of human AAA. The porcine pancreatic elastase (PPE) perfusion model was first reported by Anidjar et al. in 1990, necessitating in vivo isolation and cannulation of the abdominal aorta to administer elastase, preceding careful closure of the surgical incision. This model is technically demanding, as murine aortic isolation from the adjacent inferior vena cava and small lumbar branch vessels is difficult. Furthermore, the extent of vascular injury may differ amongst animals because of variations in perfusion pressure. Finally, careful repair of the aortotomy whilst avoiding stenosis is crucial to restore antegrade blood flow [4].

More than 20 years later, a revised version of this model in which PPE is applied to the peri-adventitial aorta was introduced by Bhamidipati et al. to surmount the intricacies related to the original perfusion model. Their model can be successfully implemented without individual vessel manipulation and consistently produces infra-renal aneurysms by Day 14 with greater inflammatory cell infiltration, decreased expression of smooth muscle protein, elastic lamina degradation, and upregulated matrix metalloproteinase activity, like that observed in other experimental AAA models [5].

The PET radiotracer [^18^F]fluorothymidine ([^18^F]FLT) is a marker of proliferative activity. We previously demonstrated increased [^18^F]FLT uptake in the angiotensin II (AngII)-induced murine model of AAA, particularly in the early phase of the model during active aneurysm growth, characterised by cell proliferation and vessel remodelling [6]. However, this radiotracer has not been examined in other preclinical models of AAA. Here, we present data on [^18^F]FLT uptake with static PET/CT imaging in the peri-adventitial PPE model of AAA.

## 2. Materials and Methods

All animal work was conducted in accordance with the UK Home Office, Animals (Scientific Procedures) Act 1986 under Project Licence P606320FB. Seven male C57BL6/J mice (Charles River, UK) were maintained at 21 °C with a 12-h light/dark cycle and 50–70% humidity in GM500 individually ventilated cages (Techniplast, Italy) and fed standard chow diet and Hydropac water *ad libitum.* All mice were provided with a housing dome and two chew sticks as environmental enrichment. At 12 weeks of age, the mice were subjected to laparotomy, and the abdominal aorta was exposed by blunt dissection. PPE (10 μL, Sigma E1250) was applied to the adventitia of the infra-renal aorta for 5 min, as per Waduud et al. [7]. Three sham-operated matched male C57BL6/J mice were used as controls; these animals underwent identical laparotomy and aortic exposure, but saline washout only.

In vivo USS was performed as per Waduud et al. using the Vevo2100 preclinical µUSS system (Visualsonics, FUJIFILM VisualSonics, Inc., Toronto, ON, Canada) with an MS-550D transducer at a 40-MHz frequency to generate three-dimensional lumen volume measurements [7]. Prior to in vivo imaging, mice were induced with 5% isoflurane/oxygen (*v*/*v*) anaesthesia before maintenance at 2% at 2 L/min. [^18^F]FLT was injected at a mean ± standard deviation dose of 9.3 ± 0.7 MBq in 100 μL of 0.9% saline solution (Aqupharm No1, Animalcare Ltd., York, UK). After 80 min to allow for biodistribution of the radiotracer, images were acquired using the Albira Si (Bruker) small-animal PET/CT scanner with animals placed prone and the field-of-view centred on the abdominal aorta. Mice were maintained at 2% anaesthesia during scanning, with temperature and respiration monitored throughout (Bruker). Following the 20-min static PET scan, a CT image was acquired for anatomical co-registration. PET/CT images were reconstructed using the maximum likelihood estimation maximisation algorithm (25 iterations), yielding voxel dimensions of 0.5 mm^3^. Ex vivo whole-organ gamma counting was performed using the Hidex gamma counter and histological staining with Ki67 (a cell proliferation marker) were performed, as per Gandhi et al. [6]. Stained aortic samples were analysed for the average proportion of Ki67-positive cells in three randomly selected regions containing proliferative cells (i.e., within the aortic wall). All quantitative analyses were performed using ImageJ 1.51k software [8]. All statistical analyses were performed using Origin 2017 software (OriginLab Corporation, Northampton, MA, USA). All graphs show the mean ± standard error of the mean (SEM). Histology, USS, and gamma counting results were analysed using two-sampled *t* tests with Welch’s correction. PET/CT results were analysed using one-way analysis of variance with post hoc Bonferroni–Holm correction. The threshold for statistical significance was set at *p* < 0.05.

## 3. Results

**Figure 1:**
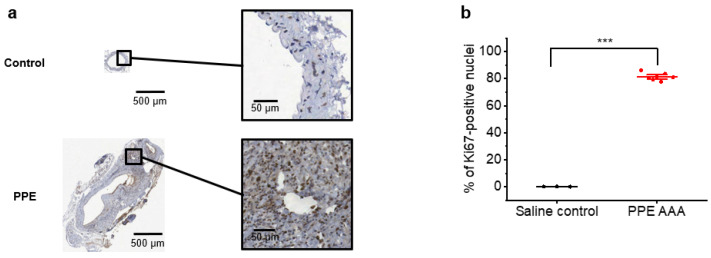
(**a**) Representative histological images of Ki67-stained aortic tissue from control and the PPE AAA model. Ki67 positivity is indicated by the brown colour, and a blue haematoxylin counterstain has been applied to aid visualisation. (**b**) Proportion of Ki67-positive nuclei in controls (*n* = 3) and in the PPE AAA model (*n* = 7). *** *p* < 0.001 on one-way ANOVA with post-hoc Bonferroni–Holm correction.

Immunohistochemistry of aortic samples from PPE AAA and sham controls revealed increased Ki67 staining in the PPE AAA aortae compared to that in sham control tissue (Figure 1a). The proportions of Ki67-positive nuclei (mean ± SEM) in the PPE AAA and control tissues were 81.4 ± 1.1 and 0.11 ± 0.01, respectively (*p* < 0.001; Figure 1b).

**Figure 2:**
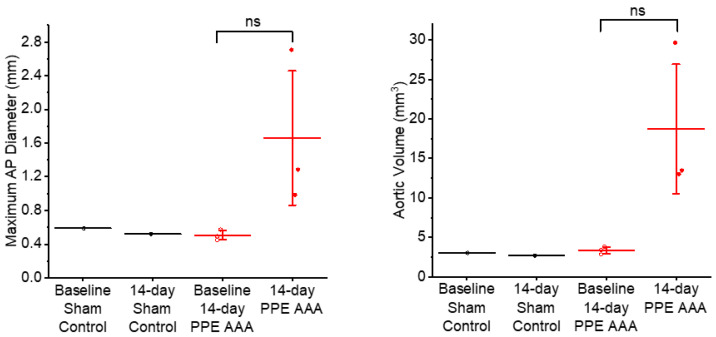
Aortic anterior–posterior diameters and volumes based on three-dimensional USS of 14-day PPE AAA vs. 14-day sham control aortae. ns: not significant on two-sample *t* test.

As expected, the three-dimensional USS images revealed significant increases in the aortic diameter (*p* < 0.05) and volume (*p* < 0.05) 14 days post-PPE application compared to baseline and sham-operated controls in the PPE AAA mice. No change in aortic size was observed in the sham controls between baseline and Day 14 (Figure 2).

**Figure 3:**
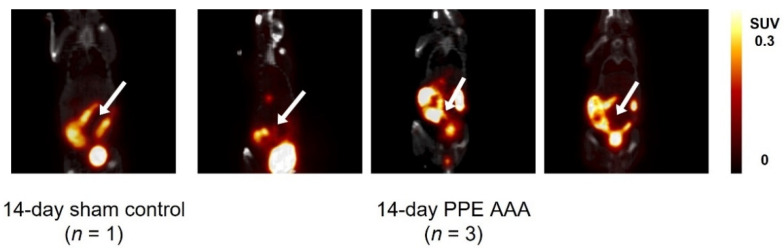
Coronal-view PET/CT images of static 14-day uptake of [^18^F]FLT 80 min post-radiotracer injection in all animals. Arrows indicate the abdominal aortic region. Colour scale bars indicate SUV thresholding to aid visualisation.

Static PET/CT images revealed that [^18^F]FLT uptake in the 14-day PPE AAA model 80 min post-injection of [^18^F]FLT was variable and noisy due to uptake in regions surrounding the abdominal aorta, such as areas of the bowel and the laparotomy wound itself. Quantification of the aortic uptake of [^18^F]FLT was therefore subject to bias and inaccuracy (Figure 3).

**Figure 4:**
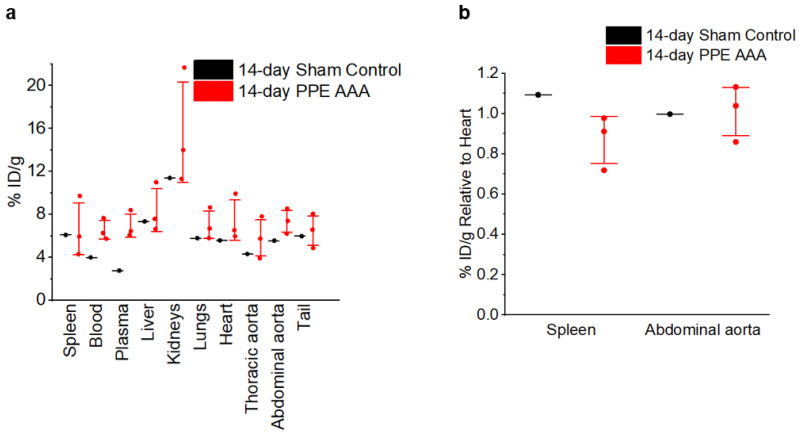
Decay-corrected ex vivo [^18^F]FLT counts per mass units. (**a**) All organs. (**b**) Uptake in the spleen and abdominal aorta normalised to uptake in the heart.

Ex vivo gamma counting revealed no significant difference in [^18^F]FLT counts in the abdominal aorta relative to the heart between the 14-day PPE AAA (*n* = 3) and 14-day sham control (*n* = 1) (Figure 4), suggesting that there was no significant [^18^F]FLT uptake in the AAA.

## 4. Discussion

The PPE-induced model of AAA is a well-established disease model that is less likely to rupture than the AngII-induced model, making it a popular choice of model for researchers [9,10]. This is the first study to investigate the uptake of [^18^F]FLT, a cell proliferation marker, in the PPE-induced murine model of AAA. We showed that [^18^F]FLT uptake is variable in this model, despite the strong Ki67 positivity and USS-based volume and diameter indicating the presence of AAA. The following methodological points must be considered in light of these findings.

The diffuse background uptake of [^18^F]FLT was not previously observed in the AngII-induced AAA model [6], leading to the suspicion that these hotspots in the abdominal wall and intra-abdominal hollow organs were related to the laparotomy required to access the aorta to deliver the PPE (or sham control procedure). Wound healing and the development of post-surgical adhesions in the abdomen might explain this increased [^18^F]FLT signal pattern. Competitive radiotracer kinetics might further account for the reduced [^18^F]FLT uptake within the aneurysm in the PPE model due to the heightened background uptake.

Nevertheless, when we performed ex vivo gamma counting of the aortic tissue and normalisation to uptake in the heart, a significantly increased signal was not observed. We have recently shown with serial USS that the peak AAA size in the PPE model occurs at approximately Day 10 post-PPE application with a decrease in size after this time point [7]. We imaged the animals 14 days post-PPE application, which is the standard time point for this model, but we may have missed the active growth phase of the model when cells are remodelling and proliferating within the aortic wall. Future studies should perform serial [^18^F]FLT PET/CT scans to precisely capture the time point at which the peak of cell proliferation occurs. Furthermore, a dynamic imaging protocol implemented at several timepoints post-AAA induction followed by appropriate kinetic analysis may yield more detailed information on radiotracer uptake, transport, and biodistribution.

## 5. Conclusions

[^18^F]FLT uptake in the PPE-induced mouse model of AAA was not robustly observed on day 14 but may be confounded by the invasive surgery to expose the aorta and timing of early proliferative activity. We anticipate that this report will be useful for researchers planning molecular imaging studies using the PPE-induced murine model of AAA.

## Data Availability

The data presented in this study are available within the present article.

## References

[1] Bell M., Gandhi R., Shawer H., Tsoumpas C., Bailey M.A. (2021). Imaging Biological Pathways in Abdominal Aortic Aneurysms Using Positron Emission Tomography. Arterioscler. Thromb. Vasc. Biol..

[2] Gandhi R., Bell M., Bailey M., Tsoumpas C. (2021). Prospect of Positron Emission Tomography for Abdominal Aortic Aneurysm Risk Stratification. J. Nucl. Cardiol..

[3] Gandhi R., Bailey M.A., Tsoumpas C. (2021). Radionuclide Molecular Imaging of Abdominal Aortic Aneurysms for Risk Stratification and Non-Invasive Therapy Assessment. Clin. Transl. Med..

[4] Anidjar S., Salzmann J.L., Gentric D., Lagneau P., Camilleri J.P., Michel J.B. (1990). Elastase-Induced Experimental Aneurysms in Rats. Circulation.

[5] Bhamidipati C.M., Mehta G.S., Lu G., Moehle C.W., Barbery C., DiMusto P.D., Laser A., Kron I.L., Upchurch G.R., Ailawadi G. (2012). Development of a Novel Murine Model of Aortic Aneurysms Using Peri-Adventitial Elastase. Surgery.

[6] Gandhi R., Cawthorne C., Craggs L.J.L., Wright J.D., Domarkas J., He P., Koch-Paszkowski J., Shires M., Scarsbrook A.F., Archibald S.J. (2019). Cell Proliferation Detected Using [^18^F]FLT PET/CT as an Early Marker of Abdominal Aortic Aneurysm. J. Nucl. Cardiol..

[7] Waduud M.A., Kandavelu P., Reay M., Paradine K., Scott D.J.A., Bailey M.A. (2021). High-Frequency Three-Dimensional Lumen Volume Ultrasound Is a Sensitive Method to Detect Early Aneurysmal Change in Elastase-Induced Murine Abdominal Aortic Aneurysm. Aorta.

[8] Rasband W.S. U. S. National Institutes of Health, Bethesda, Maryland, USA. http://ci.nii.ac.jp/naid/20000508795/en/.

[9] Sénémaud J., Caligiuri G., Etienne H., Delbosc S., Michel J.-B., Coscas R. (2017). Translational Relevance and Recent Advances of Animal Models of Abdominal Aortic Aneurysm. Arterioscler. Thromb. Vasc. Biol..

[10] English S.J., Piert M.R., Diaz J.A., Gordon D., Ghosh A., D’Alecy L.G., Whitesall S.E., Sharma A.K., DeRoo E.P., Watt T. (2015). Increased 18F-FDG Uptake Is Predictive of Rupture in a Novel Rat Abdominal Aortic Aneurysm Rupture Model. Ann. Surg..

